# Arginine-Dependent Nitric Oxide Generation and S-Nitrosation in the Non-Photosynthetic Unicellular Alga *Polytomella parva*

**DOI:** 10.3390/antiox11050949

**Published:** 2022-05-11

**Authors:** Tatiana Lapina, Vladislav Statinov, Roman Puzanskiy, Elena Ermilova

**Affiliations:** 1 Biological Faculty, Saint-Petersburg State University, 199034 Saint-Petersburg, Russia; t.lapina@spbu.ru (T.L.); st067882@student.spbu.ru (V.S.); puzansky@yandex.ru (R.P.); 2 Komarov Botanical Institute of the Russian Academy of Sciences, 197376 Saint Petersburg, Russia

**Keywords:** nitric oxide, *Polytomella parva*, S-nitrosation

## Abstract

Nitric oxide (NO) acts as a key signaling molecule in higher plants, regulating many physiological processes. Several photosynthetic algae from different lineages are also known to produce NO. However, it remains unclear whether this messenger is produced by non-photosynthetic algae. Among these organisms, the colorless alga *Polytomella parva* is a special case, as it has lost not only its plastid genome, but also nitrate reductase and nitrite reductase. Up to now, the question of whether NO synthesis occurs in the absence of functional nitrate reductase (NR) and the assimilation of nitrates/nitrites in *P. parva* has not been elucidated. Using spectrofluorometric assays and confocal microscopy with NO-sensitive fluorescence dye, we demonstrate L-arginine-dependent NO synthesis by *P. parva* cells. Based on a pharmacological approach, we propose the existence of arginine-dependent NO synthase-like activity in this non-photosynthetic alga. GC-MS analysis provides primary evidence that *P. parva* synthesizes putrescine, which is not an NO source in this alga. Moreover, the generated NO causes the S-nitrosation of protein cysteine thiol groups. Together, our data argue for NR-independent NO synthesis and its active role in S-nitrosation as an essential post-translational modification in *P. parva*.

## 1. Introduction

Nitric oxide (NO) acts as a secondary messenger with multiple functions in animals, fungi, plants, and bacteria. In plants, NO is involved in many physiological processes, such as growth and development, leaf senescence, responses to biotic and abiotic stresses, and plant–pathogen interactions, which have been reviewed previously [1,2,3,4,5].

One intriguing aspect that is not yet fully understood concerns how NO can be formed in plants. Two main pathways, reductive and oxidative, appear to explain NO production. The reductive pathway is based on the reduction of nitrites to NO. The cytosolic nitrate reductase (NR)-mediated and the mitochondrial electron transport chain-dependent reductions of nitrite to NO are the most relevant sources of NO synthesis in higher plants [6,7]. In the green alga *Chlamydomonas reinhardtii,* the complex between NR and nitric oxide-forming nitrite reductase (NOFNiR) performs reductive NO generation [8]. The oxidative pathway is well characterized in animals and is based on the oxidation of L-arginine (Arg) by Arg-dependent NO synthase (NOS) [9,10]. Among plants, only a few algae have animal-type NOS [11,12]. Interestingly, an increasing amount of evidence is in favor of the existence of NOS-like activity in plants [6]. Nevertheless, the molecular identity of the corresponding proteins remains unknown. Lastly, it has been proposed that another potential source of NO is represented by polyamines [13].

Notably, NO appears to mediate its effects in animals and plants by divergent mechanisms, and the classical NO/cGMP signaling module is not conserved in plants [6,14]. In plant cells, NO is responsible for three main post-translational modifications (PTM) of proteins such as S-nitrosation, tyrosine nitration, and metal nitrosylation [15,16]. Protein S-nitrosation is considered the most important NO-dependent signaling mechanism [13]. Here, a thiol group of protein cysteine residues is modified to the -S-NO state, and S-nitrosated proteins can alter their activity, subcellular localization, and function, all of which ultimately lead to physiological responses [1].

Compared to higher plants, the S-nitrosome of algae has been poorly studied. Recently, the S-nitrosation of proteins from *C. reinhardtii* has been reported [17,18,19]. Closely related to *C. reinhardtii*, the non-photosynthetic *Polytomella parva* possesses nuclear and mitochondrial genomes, but has lost its plastid genome [20]. The biochemical and metabolic adaptation strategies of *P. parva* in response to different nitrogen regimes are characterized by unique features [21,22]. Due to the lack of NR and nitrite reductase, this alga does not assimilate nitrate/nitrite [20,23]. Up to now, the question of whether NO synthesis occurs in the absence of functional nitrate reductase and assimilation of nitrates/nitrites in *P. parva* has not been experimentally explored. Therefore, *P. parva* is an attractive model system for exploring the evolutionary pressure to maintain NO generation in the absence of nitrates.

The present study is the first to address NO production and S-nitrosation as an NO-dependent signaling mechanism in the non-photosynthetic alga *P. parva.*

## 2. Materials and Methods

### 2.1. Algal Strain and Cultivation Conditions

*P. parva* SAG 63-3 was obtained from the SAG algal culture collection of the University of Göttingen (http://sagdb.uni-goettingen.de/, on 2 May 2018) and axenized using carbenicillin. SAG 63-3 cells were grown in medium (named REP medium) containing 20 mM tartaric acid, 40 mM ethanol as a carbon source, and 7.5 mM NH_4_Cl as a nitrogen source at pH 4.0 [24] in low light at 22 °C without shaking. To determine the growth rates, different media were used depending on the carbon source: medium with 17.4 mM acetate, pH 6.0 or pH 7.0, containing 1 mM or 5 mM arginine and REP medium containing 1 mM arginine. The number of cells was recorded microscopically with the use of a counting chamber. Four hundred cells from each sample were scored for three biological replicates. The number of viable cells was counted microscopically with the use of 0.05% (v/v) Evans blue (Dia-M, Moscow, Russia) as previously described [25]. The numbers of non-viable (stained) and viable (unstained) cells were determined. The growth rate was evaluated as a doubling of the cell number per hour.

The chemical compounds aminoguanidine (AG), N(ω)-nitro-L-arginine methyl ester (L-NAME), and DL-α-Difluoromethylornithine (DFMO) were all from Sigma-Aldrich (St. Louis, MO, USA).

### 2.2. Determination of Arginine Contents

The intracellular arginine contents were measured as described [22]. Briefly, the cells were cooled on ice just before centrifugation, washed twice at 4 °C, and then the pellet was frozen at −20 °C until further use. When the samples were thawed on ice, intracellular arginine was extracted with distilled H_2_O for 20 min at 95 °C and analyzed in supernatant after centrifugation (12,000× *g*, 5 min). The total free amino acid was measured as described [26]. Amounts of 0.1 mL of 0.2% 8-hydroxyquinoline and 0.1 mL of 2 M NaOH were added to the supernatant and the reaction mixture was incubated for 10 min on ice. After the addition of 0.1 mL of 19% sodium hypochlorite and vortexing for 30 s, the reaction was stopped by the addition of 0.1 mL of 40% urea. Absorbance was measured at 500 nm.

### 2.3. Determination of Putrescine and Ornithine and Contents

The measurement and calculations of putrescine and ornithine were conducted as previously described [27]. Briefly, 3 × 10^7^ cells were collected for these assays by gentle centrifugation at 4000× *g*, and each pellet was immediately frozen with liquid nitrogen. The metabolites were extracted and derivatized as previously reported [28]. GC-MS analysis was performed by an Agilent 6850 chromatograph (Agilent Technologies, Santa Clara, CA, USA) with an Agilent 5975 mass selective detector. Separation was performed on an Agilent J&W VF-5 ms capillary column. The helium flow rate was 1 mL min^−1^. The inlet temperature was 250 °C and the splitless mode was used. The temperature conditions of the column thermostat were as follows: initial temperature of 70 °C, increased by 6 °C per min up to 320 °C. Electron impact ionization was performed at 70 V and an ion source temperature of 230 °C. For accurate target peak identification, standards of putrescine and ornithine (Sigma-Aldrich, St. Louis, MO, USA) were analyzed. Arbitrary quantification was conducted as the normalization of the metabolite peak area by the internal standard (tricosane) peak area. Then, calibration with the putrescine and ornithine standards was conducted for absolute quantification. The analysis of the GC-MS data was processed using the PARADISE program [29] in association with the NIST MS search program and mass-spectrometer library NIST2010 (National Institute of Standards and Technology, Gaithersburg, MD, USA). Statistical processing was performed in the language environment R 4.1.0 “Camp Pontanezen” (R Core Team 2021).

### 2.4. Measurement of NO

To measure the NO levels, the cells grown in ammonium-containing medium were collected at the exponential phase of growth by centrifugation (9000× *g*, 10 min), washed with 1 mM arginine-containing medium, incubated in 1 mM arginine for the time indicated, and then exposed to 1 μM (4-amino-5-methylamino-2′7′- difluorofluorescein diacetate) dye (DAF-FM DA, Sigma-Aldrich, St. Louis, MO, USA). After 15 min of treatment, the cells were washed, resuspended in arginine-containing medium, and incubated for an additional 30 min to allow the complete de-esterification of the intracellular diacetates; then, the intracellular generation of NO was evaluated using a microplate reader CLARIOstar (BMG, Ortenberg, Germany). For some experiments, the medium was supplemented with 2 mM AG, 1 mM L-NAME, or 1 mM DFMO for 20 min before the arginine treatment. The excitation and emission wavelengths were set at 483 ± 14 and 530 ± 30 nm, respectively. Cell autofluorescence was subtracted from the total fluorescence obtained. The fluorescence levels were expressed as arbitrary units (per 10^6^ cells). Three technical replicates per condition were included on each plate, and each experiment was performed three times independently.

### 2.5. Confocal Microscopy

For the NO detection by confocal microscopy, *P. parva* cells were treated as described above. Images were acquired with a Leica TCS-SP5 confocal microscope (Leica-Microsystems, GmbH, Wettzlar, Germany) equipped with an HC PL APO 63 × oil immersion objective. Excitation was performed with a 488 nm argon laser. The signals arising from the DAF-FM DA were collected on the channel between 500 and 544 nm. The experiment was performed in triplicate.

### 2.6. Detection of Protein S-NO Groups

*P. parva* cells grown in ammonium-containing medium were collected at the exponential phase of growth by centrifugation (9000× *g*, 10 min), washed with 1 mM arginine-containing medium, and incubated in 1 mM arginine for the time indicated. For protein extraction, cells were resuspended in HENS buffer: 100 mM HEPES, pH 8.0, 1 mM EDTA, 0.1 mM neocuproine, and 1% SDS (Thermo Fisher Scientific, No. 90106, Rocksford, IL, USA), and sonicated to reduce the viscosity. The samples were centrifuged at 4 °C (15,000× g, 10 min) and proteins were precipitated from the supernatant by two volumes of acetone (−20 °C) overnight, and then the protein precipitates were washed three times with chilled 70% acetone. The proteins were resuspended in the same volume of the extraction buffer and the clear protein solutions were collected to detect the quantity of S-NO groups.

The detection of S-nitrosothiols was performed by the reduction of S-NO to -SH in the presence of ascorbate, followed by the assay of free thiol groups with 5,5′-dithiol-bis (2-nitrobenzoic acid (DTNB, Sigma-Aldrich, St. Louis, MO, USA), as described [30]. Briefly, 50 μL of 100 mM ascorbate was added to 0.9 mL samples for 1 h at 25 °C, and then the samples were treated with 50 μL of 10 mM DTNB in 75 mM phosphate buffer at pH 7. The absorbance at 412 nm was measured against control samples. The difference in the -SH quantity between the sample and control groups was used to calculate the quantity of S-NO. The measurement of free SH groups in the proteins was conducted without ascorbate treatment.

### 2.7. Protein Extraction and S-Nitrosated Protein Labeling

For the S-nitrosated protein labeling, *P. parva* were treated as described above. The cells (5 10^7^ cells ml^−1^) were collected by centrifugation (9000× *g*, 10 min) and resuspended in 4 mL of HENS buffer (Thermo Fisher Scientific, No. 90106, Rocksford, IL, USA). After centrifugation (10,000× *g*, 10 min), the protein concentration was determined by staining with a Pierse BCA Protein Assay Kit (Thermo Fisher Scientific, No. 23227, Rocksford, IL, USA). One hundred micrograms of protein in 100 µL of HENS buffer were used per sample. To block free cysteine thiols, 2 μL of 1 M sulfhydryl-reactive compound, MMTS (20 mM final concentration), was added to 100 μL of each sample and incubated for 30 min at room temperature. Proteins were precipitated by adding six volumes of pre-chilled (−20 °C) acetone and the samples were frozen at −20 °C to remove unreacted MMTS. The precipitated proteins were pelleted by centrifugation (10,000× *g,* 10 min) and resuspended in 100 μL of HENS buffer. To each 50 μL of sample, 1 μL of iodoTMT reagent (Thermo Fisher Scientific, No. 90105, Rocksford, IL, USA) was added and then 2 μL of 1 M sodium ascorbate was added to the mixture. These steps were performed in the dark. For negative control reactions, 2 μL of ultrapure water instead of sodium ascorbate was added to the samples. The reaction proceeded for 2 h at room temperature.

### 2.8. Labeled-Protein SDS-PAGE and Western Blotting

An amount of 10 μL of 5× reducing Laemmli sample buffer [31] was added to 40 μL of the labeled samples. After separation by SDS-PAGE on a 15% polyacrylamide gel, proteins were transferred to nitrocellulose membranes (Carl Roth, Karlsruhe, Germany) by semidry blotting (Trans-blot SD, Bio-Rad, Bio-Rad Laboratories, Geylang, Singap0re) and stained with Ponceau S. The blots were blocked in 5% non-fat dry milk in Tris-buffered saline solution with 0.1% Tween 20 for 1 h, prior to an incubation of 1 h in the presence of primary anti-TMT antibody (1:1000). As a secondary antibody, the horseradish peroxidase-conjugated anti-mouse IgG was used at a dilution of 1:20,000. The membranes were incubated with Super Signal West Pico Chemiluminescent Substrate for 5 min, and then the films were scanned using a Bio-Rad ChemiDocTMMP Imaging System.

### 2.9. Statistical Analysis

The values for the quantitative experiments described above were obtained from at least three independent experiments with no fewer than three technical replicates. Data represent the mean ± SE. When necessary, statistical analyses were followed by a Student’s *t* test (*p* value < 0.01).

## 3. Results

### 3.1. Effect of L-Arginine Supplementation on NO Production

**Figure 1:**
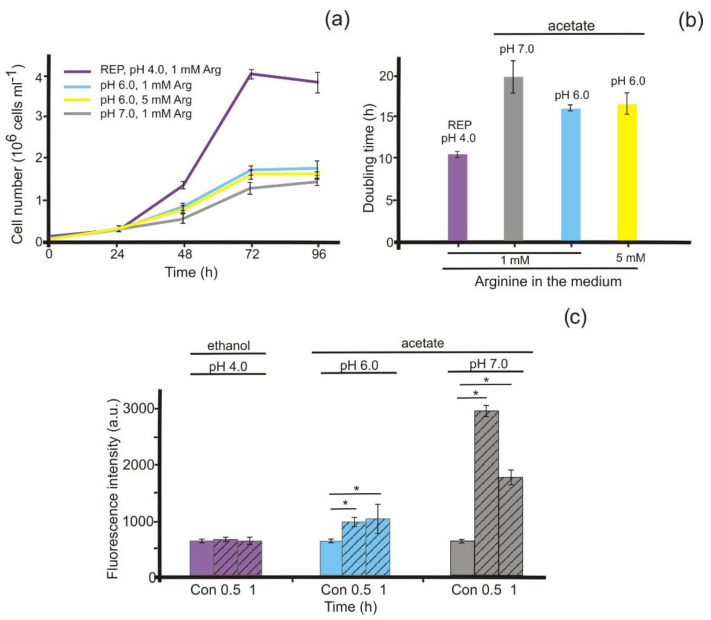
Effects of arginine on cell growth and NO production. (**a**) The growth curves were analyzed in the presence of 1 mM arginine in ethanol-containing medium at pH 4.0 (REP) or in acetate-containing media at pH 6.0 or pH 7.0. Data are presented as the mean ± SE of three independent experiments; (**b**) Doubling times calculated from the experiments shown in (**a**); (**c**) Fluorescence intensity due to intracellular NO was determined using DAF-FM DA and is expressed as arbitrary units per 10^6^ cells. Cell autofluorescence was subtracted from the total fluorescence obtained. Cells were grown in REP or acetate-containing media (**a**) and transferred to the respective medium with 1 mM arginine as a nitrogen source for 0.5 h or 1 h (shaded bars). * denotes significant differences between the control (Con) and test variants according to the Student’s *t* test (*p* value < 0.01).

We showed previously that L-arginine can be used by *P. parva* as a nitrogen source [22]. In ethanol-containing medium with 1 mM arginine as nitrogen source, the alga consistently exhibited a shorter lag phase and faster growth than in cultures that were supplemented with acetate as a carbon source instead of ethanol (Figure 1a). Additionally, in acetate-containing medium with 1 mM arginine at pH 7.0, *P. parva* demonstrated slower growth and lower final yields than it did in the medium at pH 6.0. Interestingly, the addition of 5 mM arginine to the medium did not improve the growth characteristics (Figure 1a,b) compared with *P. parva* grown in 1 mM arginine.

In *Ostreococcus tauri*, NOS is known to generate nitric oxide, and NO production is increased upon the addition of arginine [11,32]. Therefore, the potential role of arginine in NO formation in *P. parva* was analyzed. Cells incubated in ammonium-containing media showed little accumulation of NO (Figure 1c). When the alga was transferred to the arginine-containing medium with acetate, a signal increased in the cells after 0.5 h, and it was more prominent at pH 7.0 than at pH 6.0 (Figure 1c). With prolonged exposure to arginine, fluorescence decreased, but remained at a level approximately two times higher than that in the control (Figure 1c, Supplemental Figure S1). Arginine below 1.0 mM did not increase the fluorescence level.

Thus, in *P. parva* from arginine-containing medium at pH 7.0, the most reduced growth rate was observed and the highest level of NO generation (Figure 1). Moreover, in ethanol-containing medium, the addition of 1 mM arginine did not lead to an enhancement in the fluorescence level (Figure 1c).

**Figure 2:**
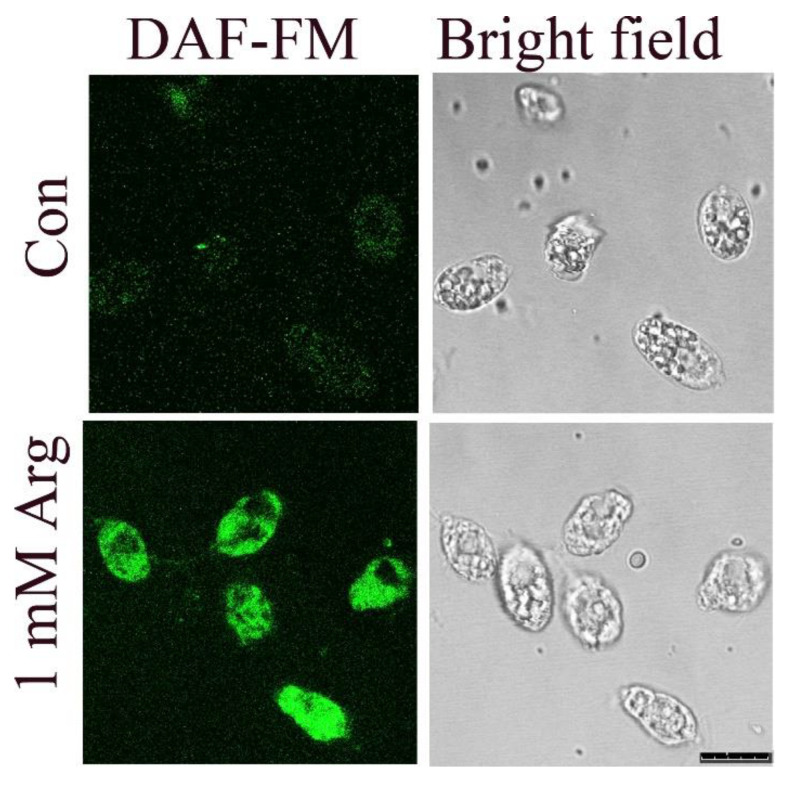
NO visualization in *P. parva* by confocal microscopy. Images of cells grown in ammonium-containing medium (Con) and then incubated with 1 mM arginine for 0.5 h. The left-hand panels show DAF-FM fluorescence (green color), while the right-hand panels show bright field images. Scale bar equals 10 μm.

These data are consistent with the images obtained by confocal microscopy (Figure 2). In ammonium-containing medium, *P. parva* showed either no signal or a very weak one in the cytosol. When the alga was transferred to the 1 mM arginine-containing medium, a green signal appeared in the cells after 30 min.

These analyses indicate that arginine had a significant impact on NO production.

### 3.2. Source of NO

**Figure 3:**
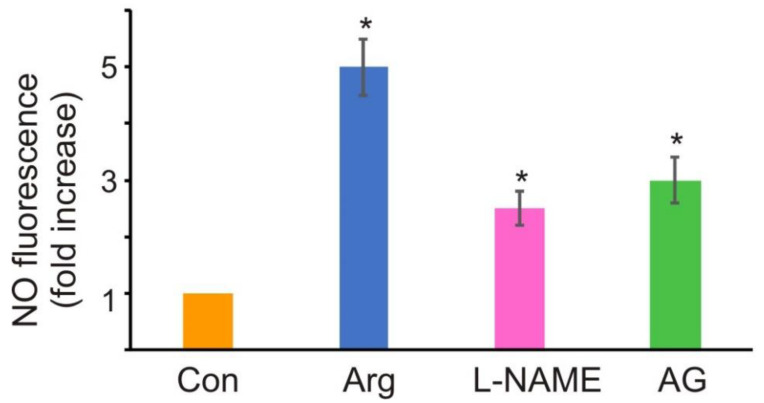
Effects of NOS inhibitors on NO synthesis. NO fluorescence was determined in *P. parva* cells treated with 1 mM Arg (pH 7.0) in the presence and absence (Con) of the NOS inhibitors, L-NAME (1 mM) or AG (2 mM), for 0.5 h. Data are expressed as the fold increase with respect to the control. Data are presented as the mean ± SE of three independent experiments. * denotes significant differences between the control (Con) and test variants according to the Student’s *t* test (*p* value < 0.01).

Since arginine is crucial for NO generation, it raises the question of the source of NO thereof. *P. parva* is an alga that lacks NR, the main enzyme involved in NO synthesis in plants [6]. Therefore, we used inhibitors of the NR-independent NO-producing pathways. The NOS-like enzyme inhibitor, L-NAME, partially affected NO production in *P. parva* after arginine treatment (Figure 3). Furthermore, NO generation was also decreased by another inhibitor of NOS, AG. 

### 3.3. Putrescine Is Formed in P. parva

In plants, arginine is not only a substrate for a NOS-like activity [33], but also a precursor of polyamines [34], which are another potential source of NO [13,35]. The centrally occurring polyamine is putrescine. The main putrescine synthesis pathway in higher plants occurs via the decarboxylation of arginine by arginine decarboxylase and the subsequent degradation of the generated agmatine [34,36]. In the second pathway, arginine is produced via ornithine formation by ornithine decarboxylase [37].

**Figure 4:**
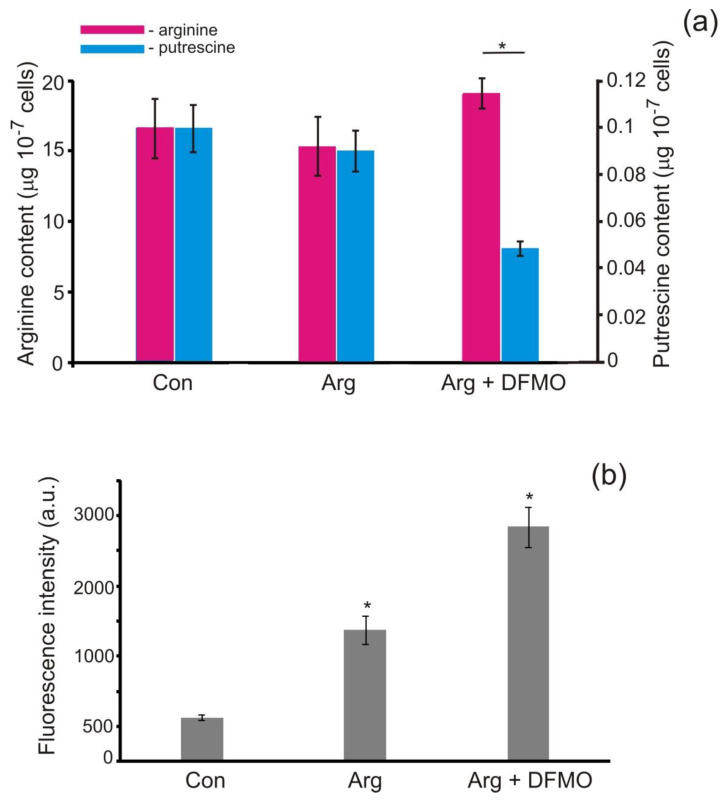
Effects of DFMO on the arginine and putrescine contents (**a**) and intracellular NO generation (**b**). Cells were grown in REP (Con) or incubated in acetate-containing medium with 1 mM arginine in the presence and absence of 1 mM DFMO for 3 h. Arginine and putrescine contents are expressed as μg in 10^7^ cells. Fluorescence intensity due to NO formation was determined using 1 μM DAF-FM and expressed as arbitrary units per 10^6^ cells. Cell autofluorescence was subtracted from the total fluorescence obtained. Means ± SEs of three biological replicates. * denotes significant differences between control (Con) and test variants according to the Student’s *t* test (*p* value < 0.01).

Although the enzymes for the synthesis of putrescine in *P. parva* have not yet been characterized [20,23], we found this polyamine in cells (Figure 4). In *C. reinhardtii*, putrescine synthesis is controlled by ornithine decarboxylase [27,38]. Ornithine, but not agmatine, was determined in our experiments (Supplementary Figure S2). These results indicate that the second route can function in *P. parva*.

The activity of ornithine decarboxylase can be inhibited by the irreversible competitive inhibitor difluoromethylornithine (DFMO) [39] We then measured the putrescine contents in the presence of DFMO (Figure 4a). Notably, after the addition of DFMO, a lower amount of putrescine was detected in the cells as compared with the control. Hence, our results suggested that putrescine production is mediated by ornithine decarboxylase in *P. parva.*

To further confirm this postulation, we determined the arginine contents. After the treatment with DFMO for 3 h, the arginine amount increased to ~24% compared to that of the non-treated cells (Figure 4a). It should be noted that increased arginine levels corresponded to increased NO production (Figure 4b). The data support the idea that the level of available arginine is likely to be one of the regulatory modes of NOS-like activity.

### 3.4. S-Nitrosation of Proteins by NO

**Figure 5:**
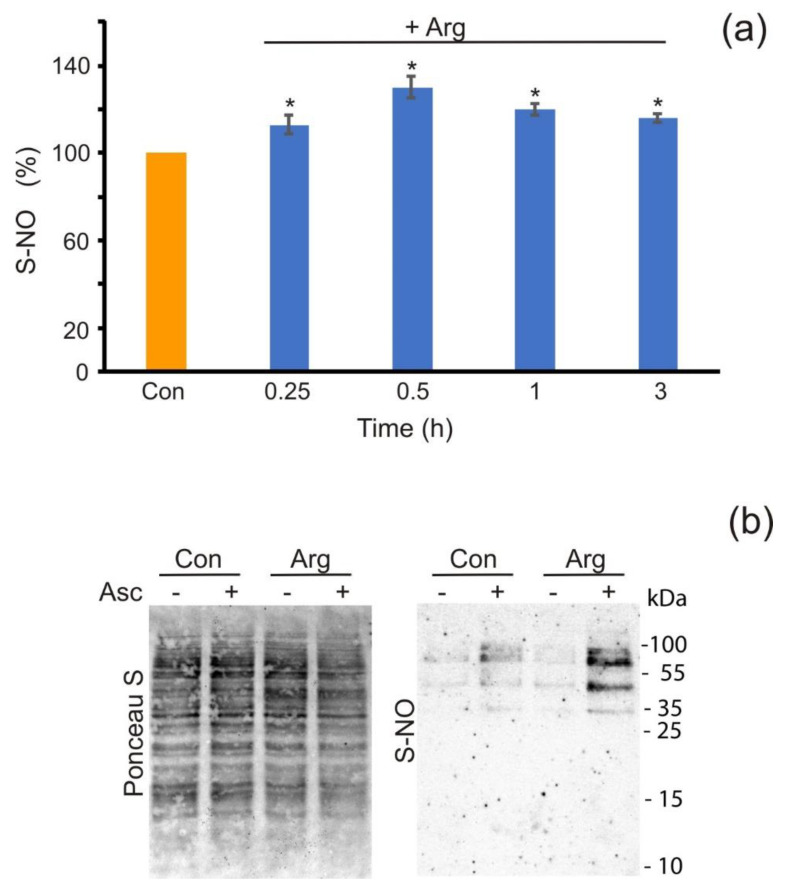
Modification of protein S-nitrosation status by arginine. (**a**) The *P. parva* samples were treated with 1 mM arginine (pH7.0) for the indicated periods. The quantity of nitrosated (−SNO) groups in proteins in the cells without added arginine represents the control (Con, set to 100%). Means ± SEs of three biological replicates. * denotes significant differences between the control and test variants according to the Student’s *t* test (*p* value < 0.01). (**b**) The samples used were without added arginine (Con) or treated with 1 mM arginine (pH 7.0) for 0.5 h. The degree of protein S-nitrosation was measured by Western blot with an anti-TMT antibody. Labeling with a non-biological iodoTMT™ reagent was performed with or without 5 mM ascorbate (Asc). Ponceau S staining was used to confirm the equal loading of the different samples.

It has been shown previously that the S-nitrosation of proteins represents one of the key mechanisms underlying NO-related signal transduction in plant cells [14]. We wondered whether the proteins undergo S-nitrosation in *P. parva* facing arginine treatment. To test this possibility, we firstly measured the levels of R−SNO groups in the proteins of cells exposed to arginine. Similar to the pattern observed for NO production (Figure 1, Supplementary Figure S1), arginine treatment induced the accumulation of R−SNO, which peaked after 0.5 h of treatment (Figure 5a). Hence, our results suggested that arginine-dependent NO generation led to increased levels of R−SNO groups.

Additionally, the Pierce S-nitrosylation Western blot kit assay was employed to determine the S-nitrosation of proteins in cells incubated with arginine. Since the fluorescence level and the quantity of −SNO groups were maximally induced after incubation with 1 mM arginine for 0.5 h (Supplementary Figure S1, Figure 5a), protein S-nitrosation by the second approach was surveyed under the same conditions. The probes were treated with the TMT reagent to label S-nitrosated proteins, and were subsequently identified using an anti-TMT antibody. Incubation with 1 mM arginine for 0.5 h produced four intense bands (between ~27 and 70 kDa) (Figure 5b). Notably, in the presence of arginine, all of the major SNO proteins were increased over the background levels; however, labeling of the ~55-kDa and ~36-kDa proteins was more prominent than that of the other species. By the same token, the S-nitrosation of these proteins in control cells was seen, but at much lower levels than those in the test extracts (Figure 5b), which is consistent with the levels of NO produced in each variant (Figure 1c). Taken together, the results indicate that S-nitrosation is a recognizable post-translational modification in *P. parva.*

## 4. Discussion

Several photosynthetic algae from different lineages are known to produce NO [1,6,14]. *P. parva* is a non-photosynthetic free-living alga, closely related to the photosynthetic *C. reinhardtii*. Although the mechanisms responsible for NO production and the NO-dependent signaling networks have been elucidated in *C. reinhardtii* [8,40,41], it remains unclear whether this messenger is produced by *P. parva*. In this work, we report original insights into NO generation and its signaling functions in cells of non-photosynthetic alga.

We demonstrate that incubation in arginine induced the rapid formation of NO in *P. parva* only when cells were transferred to an acetate-containing medium at pH 6.0 or beyond (Figure 1c), presumably because of the restriction of cell growth under these conditions (Figure 1a,b). Accordingly, treatment with arginine in ethanol-containing medium did not lead to increased NO production (Figure 1c). Additionally, here, the level of NO was inversely correlated with growth (Figure 1). Confocal microscopy also found the over-accumulation of NO in arginine-treated cells at pH 7.0 (Figure 2). These data are consistent with previous studies in plants, showing that NO synthesis is closely tied to nitrogen metabolism and depends on the availability of macronutrients [42]. We propose that arginine, or an arginine derivative, is a potential NO source.

**Figure 6:**
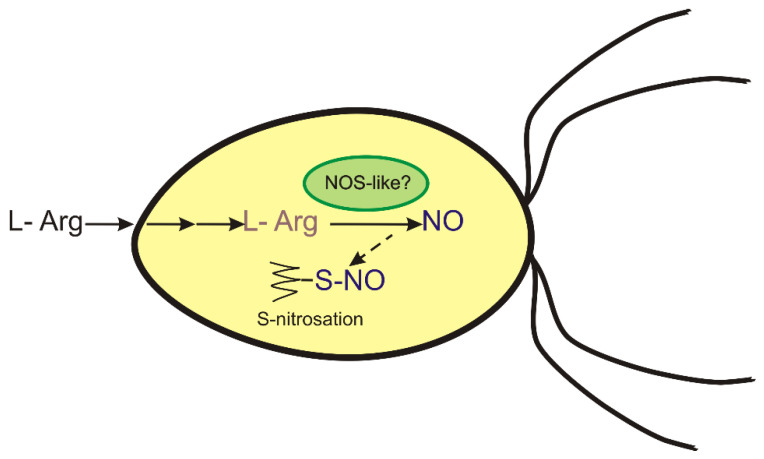
Working model for NO generation and NO signaling in *P. parva*. NO production can be achieved via an oxidative route from L-arginine. Subsequent NO signaling relies on NO-dependent post-translational modifications via S-nitrosation.

The subsequent question is how NO might be produced by *P. parva*. In photosynthetic plants, NR, mitochondrial electron transport chain, NOS-like enzymes, and putative polyamine oxidases have been suggested as sources of NO [6,7,43,44]. In contrast to embryophytes, several NOS homologs were found in algal genomes and transcriptomes [11,12,45]. In searching for NO sources, *P. parva* treatment with the mammalian NOS inhibitors L-NAME and AG was found to reduce the NO levels in cells incubated with arginine (Figure 3). Therefore, NOS-like activity may be one source involved in NO production during arginine-dependent growth in acetate-containing media (Figure 6). On the other hand, the protein(s) catalyzing this NOS-like activity in *P. parva* remains to be characterized. 

With these results, we proposed that the level of available arginine might be one of the regulatory modes of NOS-like activity. Notably, polyamine-induced NO has been suggested in higher plants to regulate embryogenesis as well as the response to drought stress [43]. Our results provide primary evidence that *P. parva* synthesizes putrescine (Figure 4a). The different putrescine contents in the control and DFMO-treated cells by GC-MS (Figure 4a) indicate that putrescine production is mediated by ornithine decarboxylase in *P. parva*. Importantly, the NO levels were markedly higher after treatment with DFMO compared with the non-treated samples (Figure 4b). In addition, the reduction of the putrescine content led to increased arginine levels, in accordance with expectations. These data confirmed the role of arginine in NO production in *P. parva.*

Another question is which protein(s) might function as NOS-like in *P. parva*. Interestingly, NOS-like activity has also been detected in various algae, such as *C. reinhardtii*, *Chattonella marina*, *Ulva compressa*, and *Symbiodinium microadriaticum* [46,47]. It is believed that plant NOS is not a canonical type as the animal NOS enzyme [33,48,49]. The possible scenario is that protein(s) with key motifs important for NOS activity could generate NO from L-arginine in *P. parva*. However, we cannot exclude the presence of an enzyme that generates NO from arginine as a residual by-product and/or the functioning of other arginine-dependent routes. Future research in this field may reveal actors to this scenario.

In plants, NO may exert its biological functions through several potential mechanisms, but it mostly occurs through S-nitrosation, the covalent and reversible binding of NO to cysteine thiol [14]. We hypothesized that this PTM would occur upon treatment with arginine in an acetate-containing medium. This does appear to be the case. We observed that, after exposure to arginine in acetate-containing medium, the nitrosation process actively developed (Figure 5). This is in agreement with the pattern of NO production (Figure 1c). Many studies have shown that S-nitrosation is a PTM by which NO can modulate protein activity [1,50,51,52]. Based on our data, we propose that the S-nitrosation of several proteins is part of the early signaling pathway leading to the NO-dependent control of *P. parva* growth in acetate-containing medium with arginine as a nitrogen source (Figure 6).

## 5. Conclusions

Collectively, our finding extends the knowledge of NO generation and signaling in plants. Apparently, it seems that during the evolution of Chlorophyta, the mechanisms of NO formation diverged in their pathways. *P. parva* is an extreme case, where NR and NiR were lost. Our main conclusion is that this unique alga generates NO via the L-arginine-dependent oxidative pathway. Moreover, establishing a role for NO in S-nitrosation provides another example of the intimate link between NO and PTM in plants, though further studies are required to characterize the proteins involved in NO synthesis and subsequent transduction of cellular signals.

## Data Availability

The data are contained within the article or Supplementary Material.

## Supplementary Materials

The following supporting information can be downloaded at: https://www.mdpi.com/article/10.3390/antiox11050949/s1, Figure S1: Time course analysis of the effect of 1 mM arginine on NO generation; Figure S2: Effects of DFMO on ornithine and agmatine contents.
