# Supplementary material for: Arginine-Dependent Nitric Oxide Generation and S-Nitrosation in the Non-Photosynthetic Unicellular Alga Polytomella parva
Source: Antioxidants (Basel). 2022 May 11;11(5):949. doi: 10.3390/antiox11050949 (PMC9138000; doi:10.3390/antiox11050949)
Supplement: Supplementary file 1 [file antioxidants-11-00949-s001.zip › antioxidants-1656725-supplementary.pdf]

Supplementary Materials:

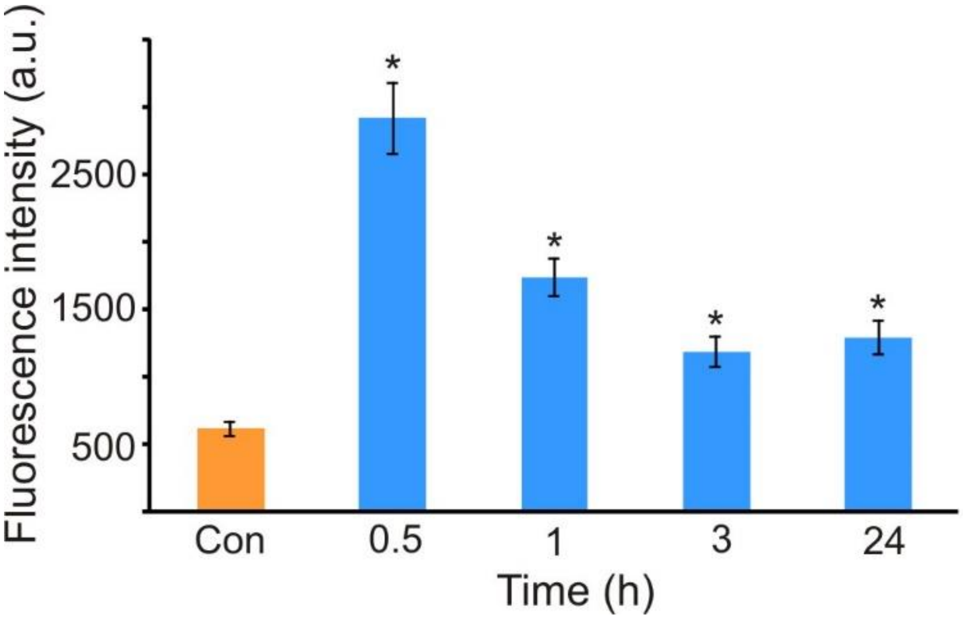

Figure 1. Time course analysis of the effect of 1 mM arginine on NO generation. Fluorescence intensity due to intracellular NO was determined using DAF-FM DA and is expressed as arbitrary units per 10^6 cells. Cell autofluorescence was subtracted from the total fluorescence obtained. Cells were grown in acetate-containing media and transferred to 1 mM arginine as a nitrogen source ***** denotes significant differences between the control and test variants according to the Student's *t* test (p value < 0.01).

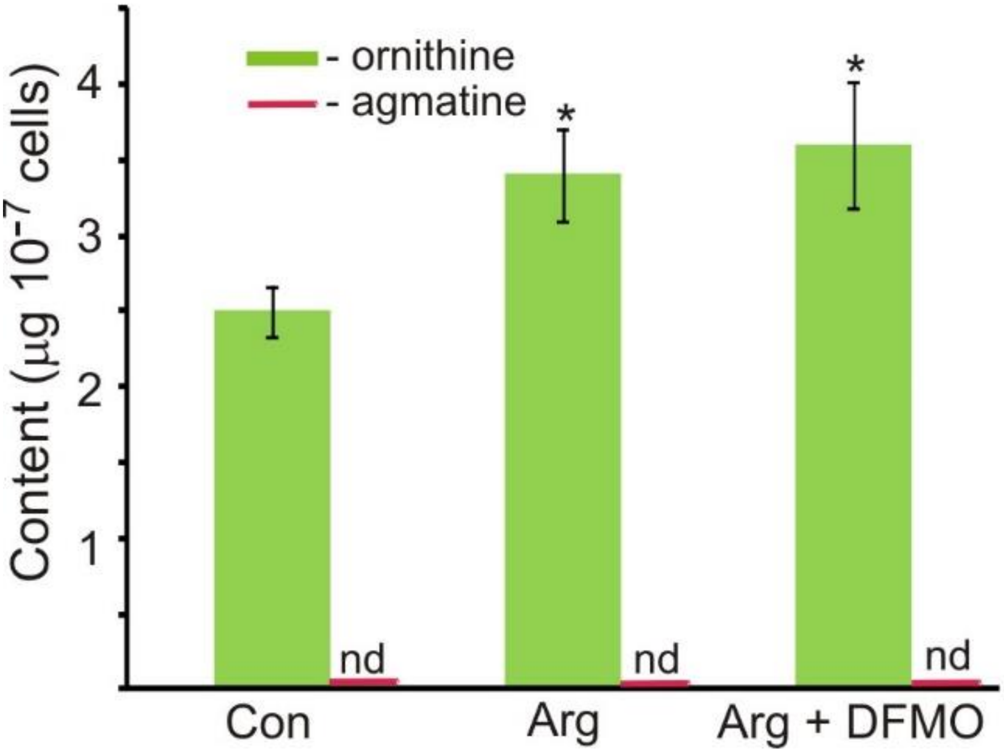

Figure 2. Effects of DFMO on ornithine and agmatine contents. Cells were grown in REP (Con) or incubated in acetate-containing medium with 1 mM arginine in the presence and absence of 1 mM DFMO for 3 h. ***** denotes significant differences between the control and test variants according to the Student's *t* test (p value < 0.01). nd, not determined.
